# Spatially-resolved analyses of muscle invasive bladder cancer microenvironment unveil a distinct fibroblast cluster associated with prognosis

**DOI:** 10.3389/fimmu.2024.1522582

**Published:** 2024-12-20

**Authors:** Chao Feng, Yaobang Wang, Wuyue Song, Tao Liu, Han Mo, Hui Liu, Shulin Wu, Zezu Qin, Zhenxing Wang, Yuting Tao, Liangyu He, Shaomei Tang, Yuanliang Xie, Qiuyan Wang, Tianyu Li

**Affiliations:** ^1^ Institute of Urology and Nephrology, The First Affiliated Hospital of Guangxi Medical University, Nanning, China; ^2^ Center for Genomic and Personalized Medicine, Guangxi Medical University, Nanning, China; ^3^ Guangxi Key Laboratory for Genomic and Personalized Medicine, Guangxi Collaborative Innovation Center for Genomic and Personalized Medicine, Nanning, China; ^4^ Department of Pathology, The First Affiliated Hospital of Guangxi Medical University, Nanning, China; ^5^ Department of Clinical Laboratory, The First Affiliated Hospital of Guangxi Medical University, Nanning, China; ^6^ Department of Urology, The First Affiliated Hospital of Guangxi Medical University, Nanning, China; ^7^ Department of Urology, Affiliated Tumor Hospital of Guangxi Medical University, Nanning, China

**Keywords:** MIBC, tumor microenvironment, spatial heterogeneity, single-cell omics, fibroblast

## Abstract

**Background:**

Muscle-invasive bladder cancer (MIBC) is a prevalent cancer characterized by molecular and clinical heterogeneity. Assessing the spatial heterogeneity of the MIBC microenvironment is crucial to understand its clinical significance.

**Methods:**

In this study, we used imaging mass cytometry (IMC) to assess the spatial heterogeneity of MIBC microenvironment across 185 regions of interest in 40 tissue samples. We focused on three primary parameters: tumor (T), leading-edge (L), and nontumor (N). Cell gating was performed using the Cytobank platform. We calculated the Euclidean distances between cells to determine cellular interactions and performed single-cell RNA sequencing (scRNA-seq) to explore the molecular characteristics and mechanisms underlying specific fibroblast (FB) clusters. scRNA-seq combined with spatial transcriptomics (ST) facilitated the identification of ligand–receptor (L–R) pairs that mediate interactions between specific FB clusters and endothelial cells. Machine learning algorithms were used to construct a prognostic gene signature.

**Results:**

The microenvironments in the N, L, and T regions of MIBC exhibited spatial heterogeneity and regional diversity in their components. A distinct FB cluster located in the L region—identified as S3—is strongly associated with poor prognosis. IMC analyses demonstrated a close spatial association between S3 and endothelial cells, with S3-positive tumors exhibiting increased blood vessel density and altered vascular morphology. The expression of vascular endothelial growth factor receptor and active vascular sprouting were significant in S3-positive tumors. scRNA-seq and ST analyses indicated that the genes upregulated in S3 were associated with angiogenesis. NOTCH1–JAG2 signaling pathway was identified as a significant L–R pair specific to S3 and endothelial cell interactions. Further analysis indicated that YAP1 was a potential regulator of S3. Machine learning algorithms and Gene Set Variation Analysis were used to establish an S3-related gene signature that was associated with the poor prognosis of tumors including MIBC, mesothelioma, glioblastoma multiforme, lower-grade glioma, stomach adenocarcinoma, uveal melanoma, kidney renal clear cell carcinoma, kidney renal papillary cell carcinoma, and lung squamous cell carcinoma.

**Conclusions:**

We assessed the spatial landscape of the MIBC microenvironment and revealed a specific FB cluster with prognostic potential. These findings offer novel insights into the spatial heterogeneity of the MIBC microenvironment and highlight its clinical significance.

## Introduction

Muscle-invasive bladder cancer (MIBC) is a malignancy of the urinary system, which is characterized by a high degree of heterogeneity, recurrence, and metastasis, leading to a poorer prognosis (1). The tumor microenvironment (TME) is considered an important determinant in cancer biology, tumor progression, and therapeutic responses (2). Previous studies have reported that the interactions between tumor cells and TME are crucial for tumor development and a significant driving force in tumor deterioration (3, 4). Fibroblasts (FB) represent an important component of TME. The stroma of normal bladder tissues contains quiescent or resting FB (5, 6). FB is activated during neoplastic cell invasion for the reconstruction of the surrounding environment (7). Then, the invaded cancer cells interact with novel environments, creating conditions conducive to the survival and migration of tumor cells (8). Cancer cells interact with neighboring cells and non-cellular components in the TME, influencing tumor growth, invasion, metastasis, and the efficacy of treatment (8). TME-related targeted therapies have recently garnered increasing attention (2). Nevertheless, the underlying reason for poorer prognosis in bladder cancer patients following the invasion of tumor cells into the muscular layers, and whether this is associated with the TME, remains unknown. Hence, investigating the spatial characteristics of the MIBC microenvironment and the spatial interactions between cells may reveal important factors driving tumor progression.

Recent advancements in spatial omics offer a new perspective, allowing the analysis of *in-situ* expression profiles and spatial distributions of TME components (3, 9). By describing cellular interactions and neighborhood patterns based on a spatial perspective, the communication networks within the TME were discovered (10). These analyses facilitate the identification of biomarkers, modulation of anti-tumor immunity, and formulation of targeted therapies for different cancers including colorectal (11), breast (12), bladder (13), and lung cancer (14). Imaging mass cytometry (IMC) has become an important tool in TME study (15). IMC allows the concurrent identification of numerous protein expressions in tissue sections, providing critical spatial data that allows for the detailed characterization of the TME’s complex spatial architecture (16).

In this study, we collected 40 MIBC tissue sections. IMC was used to determine the protein expression of 185 regions of interest (ROIs), which included 100 tumor regions (T), 62 leading-edge regions (L), and 23 nontumor regions (N). We found that the spatial structural characteristics changed from N to L to T regions. Through the in-depth analysis of the components in N, L, and T regions, four different FB clusters with unique phenotypes were found. Each of the FB clusters showed specific spatial distribution patterns. Notably, we found that a specific FB cluster (S3) is associated with abnormal angiogenesis and poor prognosis. Single-cell RNA-seq (scRNA-seq) and spatial transcriptome (ST) analyses verified that S3 interacted with endothelial cells via the receptor–ligand (L–R) pair, NOTCH1–JAG2. Lastly, based on the molecular characteristics of S3, we identified an S3-related prognostic signature, which can provide prognostic insights into patients with cancers. Altogether, the findings of this study provide novel insights into the spatial diversity from N to L to T regions, offering evidence to determine the diagnostic and therapeutic targets of MIBC.

## Materials and methods

### Patients and samples

Forty paraffin histopathological sections were obtained from the pathology department of the First Affiliated Hospital of Guangxi Medical University. All participants had not received any tumor therapy before enrolment, and the diagnosis of MIBC was confirmed by two experienced pathologists. The N, L and T region were delineated by the pathologist. Written informed consent was provided by all participants. This study was approved by the ethics committee of the First Affiliated Hospital of Guangxi Medical University (2024-E443-01). The following cohort were used for pan-cancers analysis: TCGA-BLCA, TCGA- MESO, TCGA- GBM, TCGA-LGG, TCGA-STAD, TCGA-UVM, TCGA-KIRC, TCGA-KIRP, TCGA-LUSC, GSE32894, GSE188715. The bulk RNA-seq data and clinical information of TCGA datasets can be downloaded from https://portal.gdc.cancer.gov/. As for GEO datasets, they can be found at https://www.ncbi.nlm.nih.gov/geo/.

### Imaging mass cytometry analysis

IMC was performed as previously described (17). Briefly, after dewaxing the tissue sections (thickness of 5µm), antigen retrieval was performed using Tris-EDTA antigen retrieval solution (FC16FA0005, Sangon Biotech (Shanghai) Co.,Ltd.,China). The sections were then blocked with a 3% BSA solution at room temperature and incubated overnight at 4°C with antibody cocktail, as shown in **Supplementary Table S1**. The next day, the sections were stained with Cell-IDTM Intercalator-Ir (Fluidigm) for DNA and subjected to analysis.

The MCD viewer software was used to open the files generated by the IMC system, and 16-bit OME-TIFF files were exported. The 16-bit OME-TIFF files were imported into the CellProfiler software, where channel information was set and signal intensities of each channel were extracted. The DNA (Ir191 and Ir193) and cell surface markers were used to identify the cell nuclei and cell boundaries, respectively. Single cells were then segmented and TIFF files were exported. The TIFF files exported from CellProfiler were imported into the HistoCAT software. The phenograph algorithm was applied for clustering, while the tSNE algorithm was used for high-dimensional data reduction. Neighbor analysis was employed to examine the interactions between different cell clusters. The immune cells and stromal cells were gated in cytobank (https://community.cytobank.org) by specific markers: stromal cell (Collagen I, vimentin), immune cell (CD45), B cell (CD20), macrophage (CD68), CD8+ T cell (CD3, CD8) and CD4+ T cell (CD3, CD4).

During cell division, the X/Y coordinates of each cell type were determined. Similarly to previously reported methods (18), we utilize the euclidean distance to calculate the closest distance between each cell and different cell types. On a per-ROI basis, we then computed the average closest distance between cell types within each ROI. Cellular neighborhood identification: For the 1,303,975 cells in our experiment, we set the window size to 26, consisting of one central cell and its 25 nearest neighbors, determined by euclidean distance in X/Y coordinates. We chose a 25-cell radius as a rough approximation, where the central cell distance in each direction is visually determined to be a good indicator of local functional activity.

### Single-cell RNA sequencing analysis

The scRNA-seq data from eight bladder cancer patients were downloaded from the cited literature (19). As a quality control measure, we excluded sequenced cells that had less than 200 detected genes and more than 10% mitochondrial genes. Additionally, genes expressed in fewer than three cells were also excluded. We used the FindClusters function in Seurat (version: 4.0.1) to identify 35 clusters and visualize them using a t-distributed stochastic neighbor embedding (t-SNE) plot. Cluster-specific genes were used to annotate cell types with classic markers described in previous studies (19): tumor epithelial cells (EPCAM+/KRT17+); endothelial cells (PECAM1+/ENG+); fibroblasts (COL1A1+/RGS5+); myeloid cells (LYZ+/CD68+); T cells (CD3D+/CD2+); B cells (CD79A+/MZB1+); and mast cells (TPSAB1+/CPA3+).The “ComplexHeatmap” R package was used to visualize the average expression values of marker genes for each cell cluster.

For the analysis of interactions between cell types, we extracted the expression profiles of each cell type from eight bladder cancer tissues. The expression matrix, along with cell type metadata, was used as input for running cellphonedb with the statistical analysis method in Python. This study detected statistically significant interactions (p < 0.05) between receptors and ligands by comparing them against a curated L-R database. The “ktplots” R package was used to visualize the significant L-R pairs identified by cellphonedb. Transcription factor prediction of S3 up-regulated gene was performed by BART, an online analytical tool (http://bartweb.org/).

### Spatial transcriptome analysis

Bladder cancer ST data can be obtained from published literature (13). The “SpaCET” R package was utilized to compute the proportion of tumor cells and other cell types for each spot. Spots with more than 60% tumor cells, FB, or endothelial cells were designated as tumor region, FB region, or endothelial region, respectively (20). We estimated S3 enrichment in spots by scoring each spot of the ST data using S3 up-regulated genes. Positive L-R spots need to meet the following criteria: 1. They should be located at the boundary between the FB and endothelial region. 2. The ligand and receptor should be queryable in the cellponedb database. 3. The spot should express both the corresponding ligand and receptor genes simultaneously.

The gene sets used for ST scoring can be obtained from the GSEA official website (https://www.gsea-msigdb.org/gsea/downloads.jsp). The GSVA package (version: 1.38.2) was employed to calculate the enrichment score for each spot.

### Establishment of FCS3S prognostic prediction system

Based on the expression of specific markers (Collagen1, CD90, Vimentin, and α-SMA), FB can be classified into FB S3, FB S4, and FB SX. “FindMarkers” was utilized to perform differential gene expression analysis for FB subtypes, with logFC. threshold >= 0.25 and *p*_val_adj < 0.05 considered as significantly differentially expressed genes. Further filtering of the characteristic genes specific to FB S3 was performed using xgboost.

### Statistical analysis

Statistical analysis was conducted using two-sided Student’s t-test and Kruskal-Wallis rank sum test. A significance level of *p*<0.05 was considered statistically significant. Kaplan-Meier analysis was used to estimate the overall survival curves of patients with MIBC, and the log-rank test was employed to compare the curves. GraphPad Prism 8 software was utilized for statistical analysis.

## Results

### Spatial heterogeneity of the tumor microenvironment in MIBC

**Figure 1:**
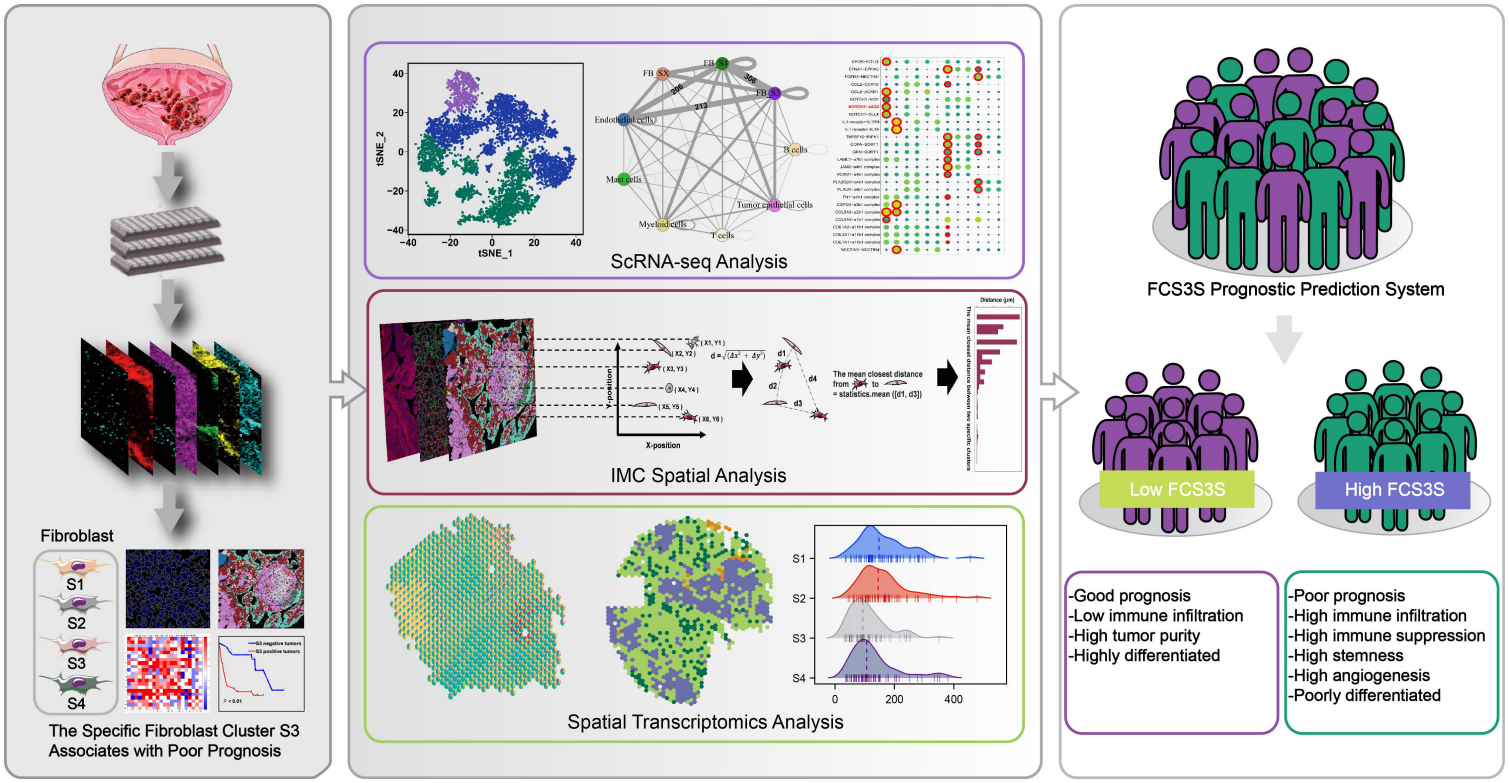
Imaging mass cytometry (IMC) with a panel of 33 TME-related markers was employed to reveal the spatial structure of microenvironment in tumor core region (T), leading-edge region (L) and nontumor regions (N). Combining with IMC analysis, scRNA-seq analysis and spatial transcriptomics (ST) analysis, we have identified a specific fibroblast cluster, denoted as S3, which was associated with poor prognosis.

**Figure 2:**
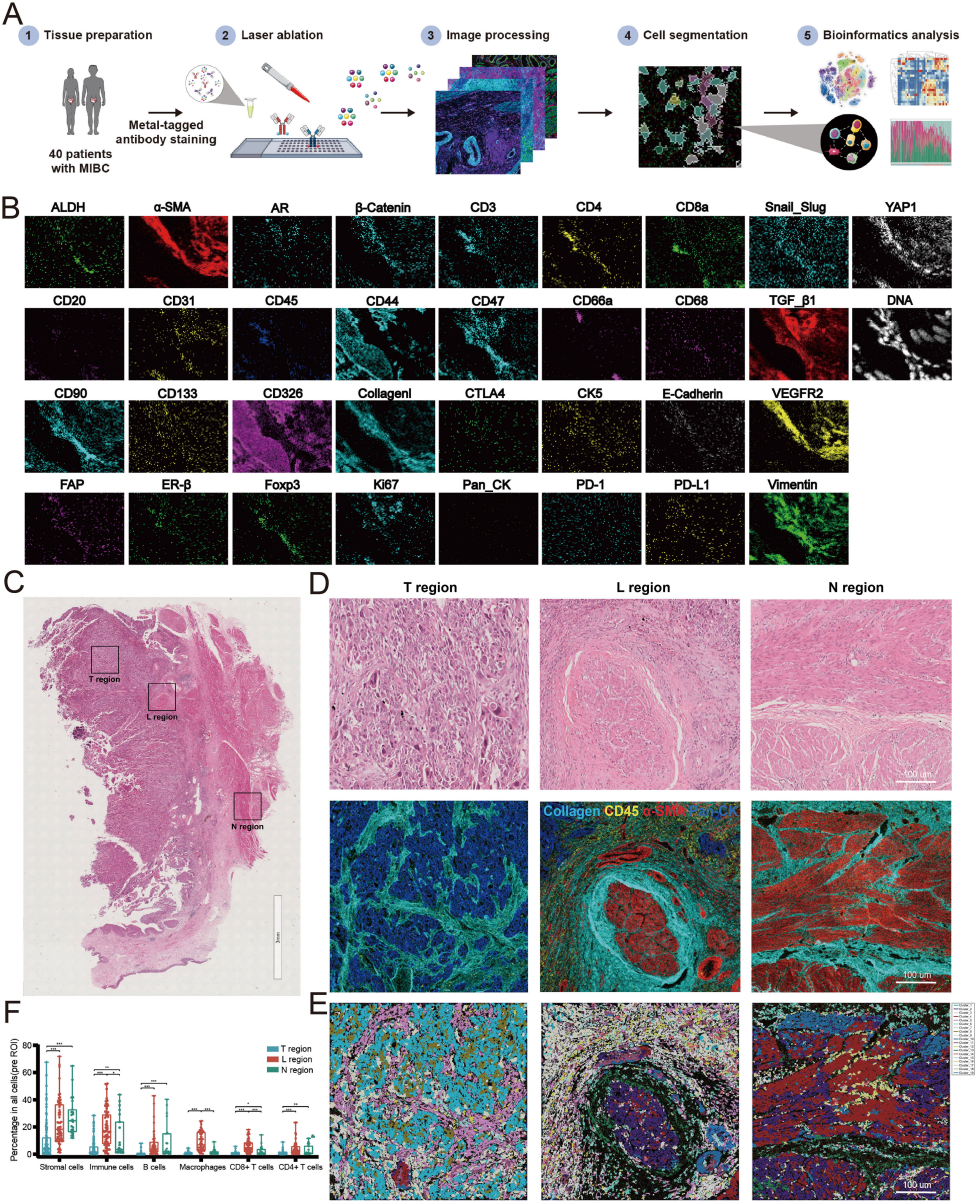
Establishment of imaging mass cytometry (IMC) analysis for MIBC microenvironment. **(A)** Workflow of IMC analysis. **(B)** Representative IMC images from a region of interesting (ROI) with 33 markers. **(C)** H&E staining diagrams of N, L and T regions. **(D)** IMC images of the corresponding H&E diagrams. **(E) **Spatial distribution images of clusters in N, L and T regions. Different colors represented different types of clusters. **(F)** Relative frequency of cells in N, L and T regions. (*P<0.05, **P<0.01, ***P<0.001).

To explore the complex spatial characteristics of the MIBC microenvironment and enhance our understanding of its role in promoting metastasis. We performed IMC with a panel of 33 TME-associated markers to detect 100 tumor core regions (referred to as T), 62 leading-edge regions (referred to as L, the boundary of tumor cell invaded into muscular layer), and 23 nontumor regions (referred to as N) from paraffin-embedded tissue sections of 40 patients with MIBC, yielding 6,105 high-quality images (**Figure 1** and **Supplementary Figure S1**). Through cellular identification and segmentation of these images, we obtained single-cell expression profiles of 33 proteins (**Figures 2A, B**). Segmentation obtained data from 185 ROIs, ranging from 1562 to more than 10,000 cells per ROI (median 7317 cells/ROI; **Supplementary Figure S2**).

We found that the spatial structure of N, L, and T regions showed significant heterogeneity (**Figures 2C, D**). By cell clustering and spatial reconstruction, we found that cells with similar phenotypes cluster nearby within the T and N regions. On the other hand, cells in the L region exhibit a more disorganized distribution, suggesting a more complicated microenvironment in the L region compared with that in the T and N regions (**Figure 2E**). The deep analysis using Cytobank (an online analytic platform) revealed that the frequencies of stromal cells decreased from N to L to T regions progressively. The frequencies of immune cells including B cells, macrophages, CD4+ T cells, and CD8+ T cells increased in the L region than in the N and T regions (**Figure 2F**). Altogether, these results suggest that the microenvironments of the N, L, and T regions are spatially heterogeneous in MIBC.

### The spatial characteristics of microenvironmental components exhibit regional diversity

**Figure 3:**
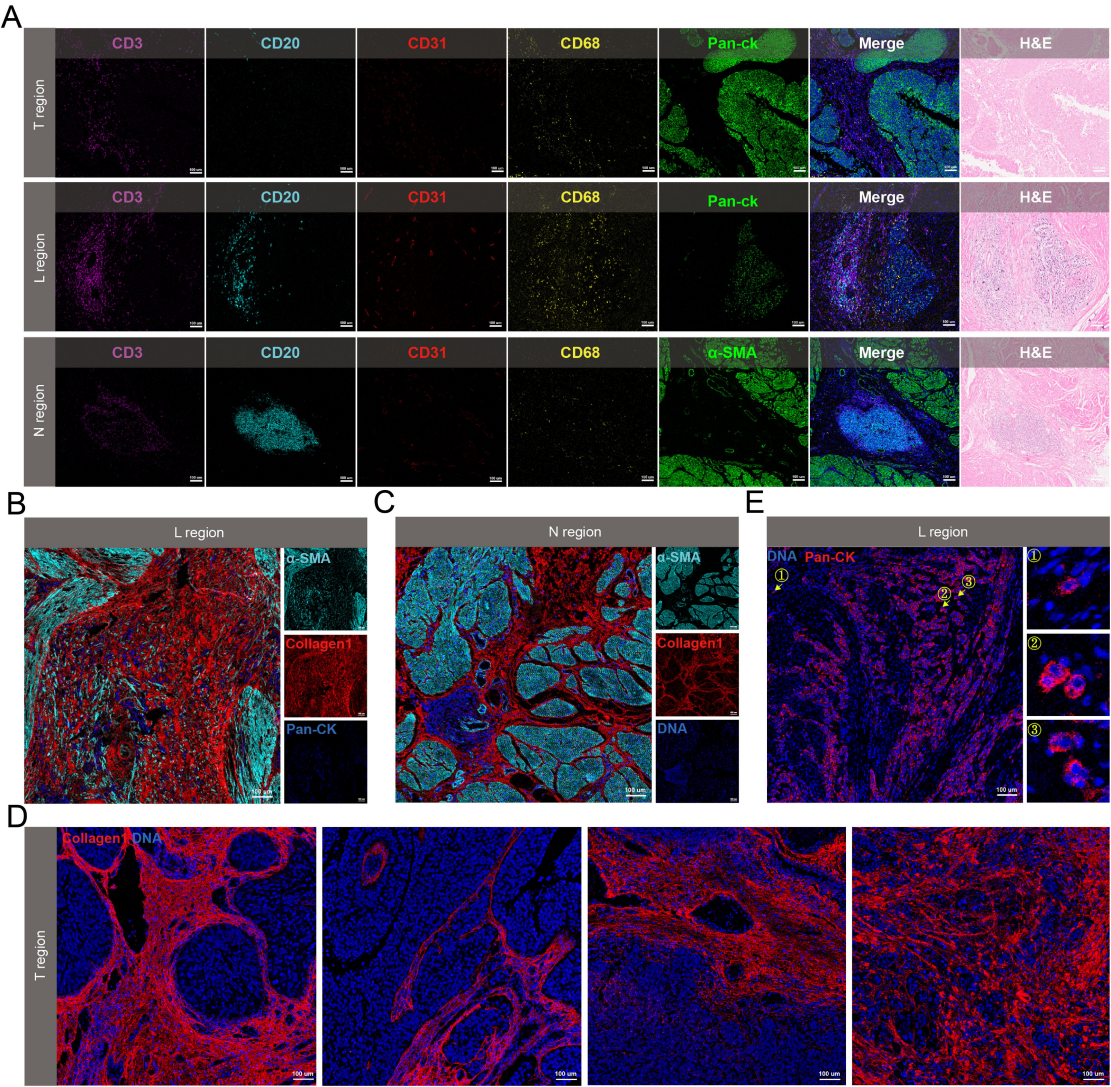
Spatial heterogeneity of the microenvironment in N, L and T regions. **(A)** Spatial distribution of immune cells in the three regions. **(B-D)** Collagen morphology in L region **(B)**, N region **(C)** and T region **(D)**. **(E)** Representative IMC images of tumor budding cells. The arrow referred to the tumor budding cells.

Subsequent analyses were performed to determine the spatial heterogeneity of the microenvironment in N, L, and T regions. In the N region, numbers of B cells were recruited, whereas T cells and macrophages were less prevalent. The L regions displayed a higher density of both B cells and T cells, which were positioned near each other, with macrophages more dispersed near the tumor cells. The T regions showed a decreased number of immune cells, which were primarily located in the stromal areas rather than infiltrating the cancer nests (**Figure 3A**). The collagen structures in these regions also exhibited regional heterogeneity. The L regions were characterized by disordered collagen configurations, and some collagen appeared as dots (**Figure 3B**). Collagen was observed as curved structures in the N regions, loosely encasing small segments of muscle tissue (**Figure 3C**). Within the T regions, the following four unique types of collagen structures were identified: curved collagen encapsulating the tumor nests, parallel collagen aligning along the cancer nest margins, directionally arranged collagen, and disorderly arranged collagen with unclear boundaries relative to the tumor (**Figure 3D**). Additionally, tumor budding cells were found in the invasive region, which were dispersed within the tumor stroma as single cells or small clusters (**Figure 3E**).

### Specific fibroblast cluster serves as a significant prognostic factor of MIBC

**Figure 4:**
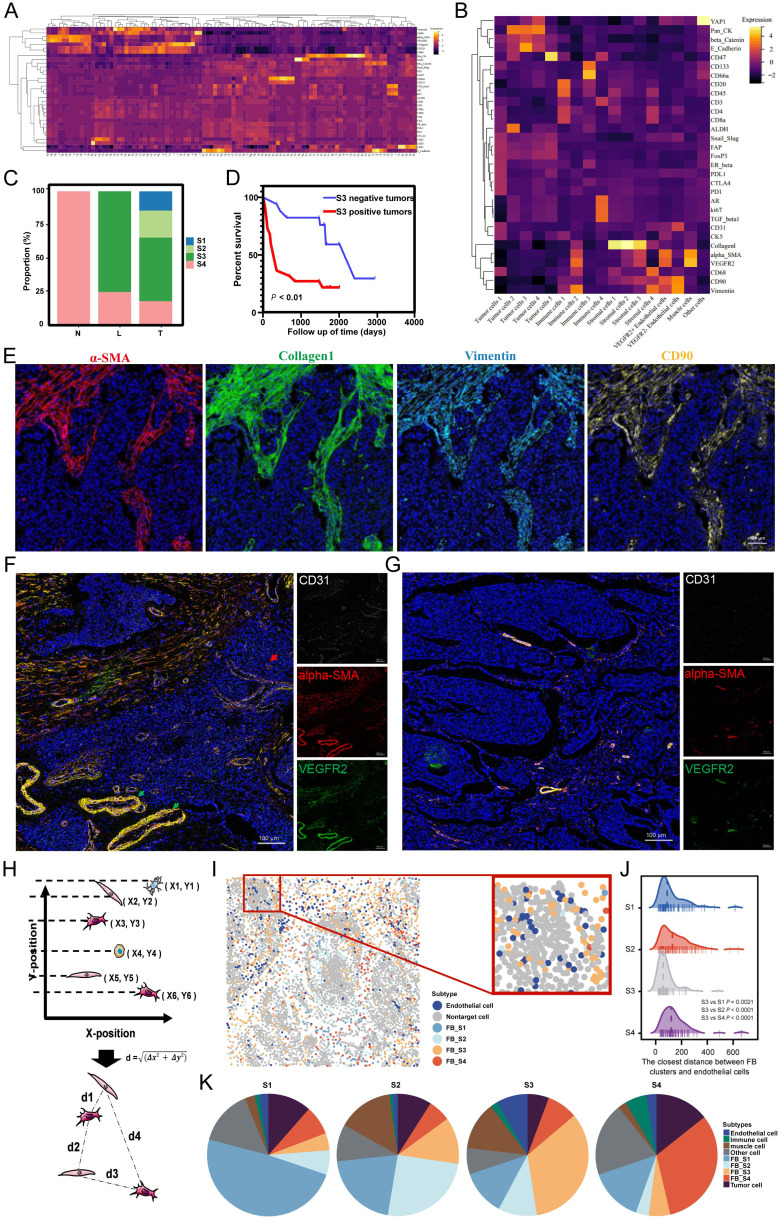
**(A)** The heatmap of the cell clusters from 185 ROIs. **(B)** The heatmap of merged cell cluster. **(C)** Distribution of four fibroblast clusters in N, L and T regions. **(D)** S3 was associated with poor prognosis. **(E)** Overlaid pictures of α-SMA (red), Collagen I (green), Vimentin (cyan) and CD90 (yellow) to identify the S3. **(F, G)** The representative images of vascular distribution in S3-positive tumor **(F)** and S3-negative tumor **(G)**. **(H)** The calculating principle of cell distance. **(I)** S3 was near to endothelial cells. **(J)** The closest distance between FB clusters and endothelial cells **(K)** Distribution of neighbor components in four FB clusters.

To explore the important components of the TME involved in the recurrence and metastasis of MIBC, we clustered cells from 100 T regions, 62 L regions, and 23 N regions into 100 cell clusters with distinct phenotypes (**Figure 4A**). Then, we consolidated these 100 clusters into 17 groups based on the expression of specific markers for tumor cells, immune cells, stromal cells, vascular endothelial cells, and muscle cells. Among these were the following four unique FB clusters: FB S1 (α-SMA− Collagen I+ Vimentin- CD90+), FB S2 (α-SMA+ Collagen I+ Vimentin- CD90+), FB S3 (α-SMA+ Collagen I+ Vimentin+ CD90+), and FB S4 (α-SMA- Collagen I+ Vimentin+ CD90+) (**Figure 4B**). These FB clusters exhibited unique spatial distributions. The L regions predominantly contained two types of FB, with nearly 75% being FB S3, referred to as S3 hereafter. Furthermore, all four FB clusters were present in the T regions, whereas the N regions exclusively contained FB S4 (**Figure 4C**).

The L regions, a critical boundary for tumor invasion and metastasis, emerged as the most dynamic area, with S3 as the predominant FB type. This suggests that S3 exhibits high activity and functional characteristics, facilitating tumor progression. Survival analysis showed that MIBC patients with S3-positive tumors had shorter overall survival than those with S3-negative tumors (**Figures 4D, E**), indicating a crucial role of S3 in MIBC progression. Investigating the TME heterogeneity between S3-positive and S3-negative tumors, we found that S3-positive tumors exhibited a higher density of blood vessels, which included more extensive and severely distorted blood vessels with abnormal dilation. Furthermore, these tumors showed high expression of VEGFR on vessel walls and outward expansion (**Figures 4F, G**), suggesting that S3 may contribute to abnormal angiogenesis. Subsequent investigation using IMC spatial analysis determined the interactions between S3 and endothelial cells. By evaluating spatial coordinates, we determined the distances from each FB cluster to the nearest endothelial cells and found that the proximity of S3 to endothelial cells was shorter compared with other FB types (**Figures 4H–J**). Neighbor component analysis among the four FB clusters showed that endothelial cells contained 2.38%, 1.43%, 8.39%, and 2.79% of the neighbors for FB S1, S2, S3, and S4, respectively (**Figure 4K**). These findings reveal an important close interaction between S3 and endothelial cells, highlighting the significant effect of S3 on MIBC angiogenesis.

### ScRNA-seq analysis reveals the association between fibroblast cluster S3 and angiogenesis

**Figure 5:**
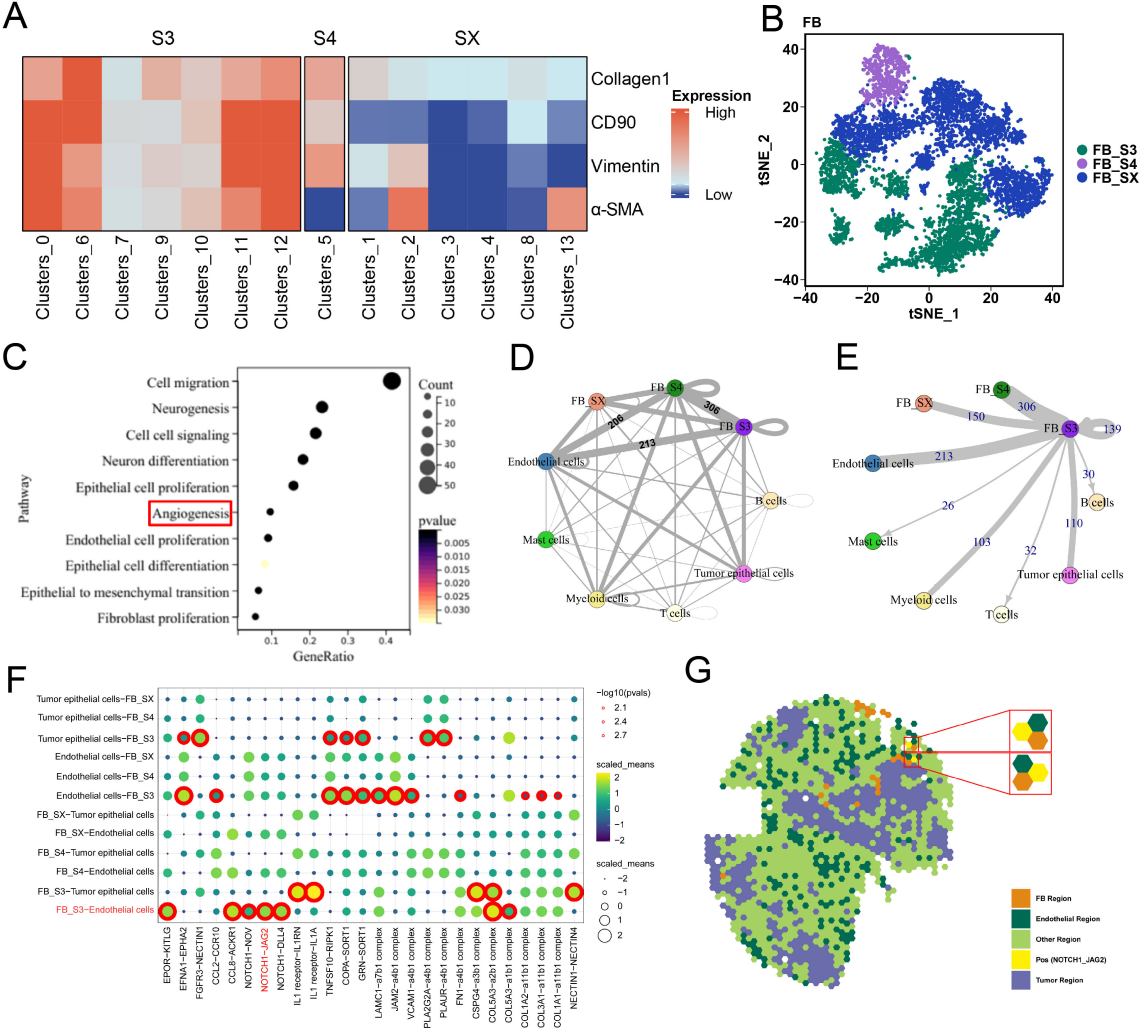
ScRNA-seq analysis revealed the molecular mechanism of S3. **(A)** The heatmap of four stromal cell markers in FB clusters. **(B)** tSNE plot of three kinds of FB clusters. **(C)** Gene ontology (GO) analysis showed that the differentially expressed genes (DEGs) of S3 and other FB clusters enrich in angiogenesis and other cancer-promoting pathways. **(D, E)** Circle plots showing S3 shared more ligand–receptor with other cells, when compare with other FB clusters. **(F)** ligand–receptor pairs between FB clusters and endothelial cells. **(G)** Spatial transcriptome analysis showed that NOTCH1 and JAG2 were spatially colocalized.

To elucidate the mechanisms underlying S3, we analyzed publicly available scRNA-seq data from eight bladder cancer samples. Through cell clustering and annotation, we found 35 unique cell clusters characterized by their specific markers, which included B cells, endothelial cells, tumor epithelial cells, fibroblasts, mast cells, myeloid cells, and T cells (**Supplementary Figures S3A, B**). Subsequent refinement of our analysis by reclustering the fibroblasts yielded 14 fibroblast clusters (**Supplementary Figure S3C**). Using the expression of stromal cell-specific markers, including Collagen 1, α-SMA, Vimentin, and CD90, we identified the previously characterized FB clusters S3 and S4 from IMC analysis and labeled the remaining FB clusters as FB SX (**Figures 5A, B**). Differential expression analysis showed unique molecular profiles among these FB types (**Supplementary Figure S3D**). The gene expression upregulated in S3 was enriched in pathways related to angiogenesis, cell migration, epithelial cell proliferation and differentiation, endothelial cell proliferation, and fibroblast proliferation (**Figure 5C**). Analysis of cellular communication among clusters via single-cell L–R interaction showed that S3 engaged in more L–R pair interactions with other cells compared to FB S4 and SX. Specifically, S3 exhibited a significantly higher number of L–R pairs with endothelial cells, showing strong communicative activity (**Figures 5D, E**). These findings indicate that S3 maintains active communication with different cell types and also engages in close interactions with endothelial cells.

### The fibroblast S3 interacts with endothelial cells by L–R pair NOTCH1–JAG2

To elucidate the mechanisms between S3 and endothelial cells, we analyzed L–R pairs unique to S3 and endothelial cells (**Figure 5F**). While scRNA-seq offers insights into the cell–cell interactions based on L–R gene expression, it does not account for cell localization. Spatial transcriptomics (ST) analysis, which profiles spatial gene expression within tissues, allows the detection of cell–cell interactions across different regions. By analyzing publicly available ST data from bladder cancer, we identified specific regions corresponding to tumor cells, FB, and endothelial cells. Furthermore, the L–R pair NOTCH1–JAG2 was co-located in the endothelial spots adjacent to the FB region, indicating that S3 may predominantly interact with endothelial cells through the NOTCH1–JAG2 pathway (**Figure 5G**).

### The fibroblast S3 is potentially regulated by YAP1

**Figure 6:**
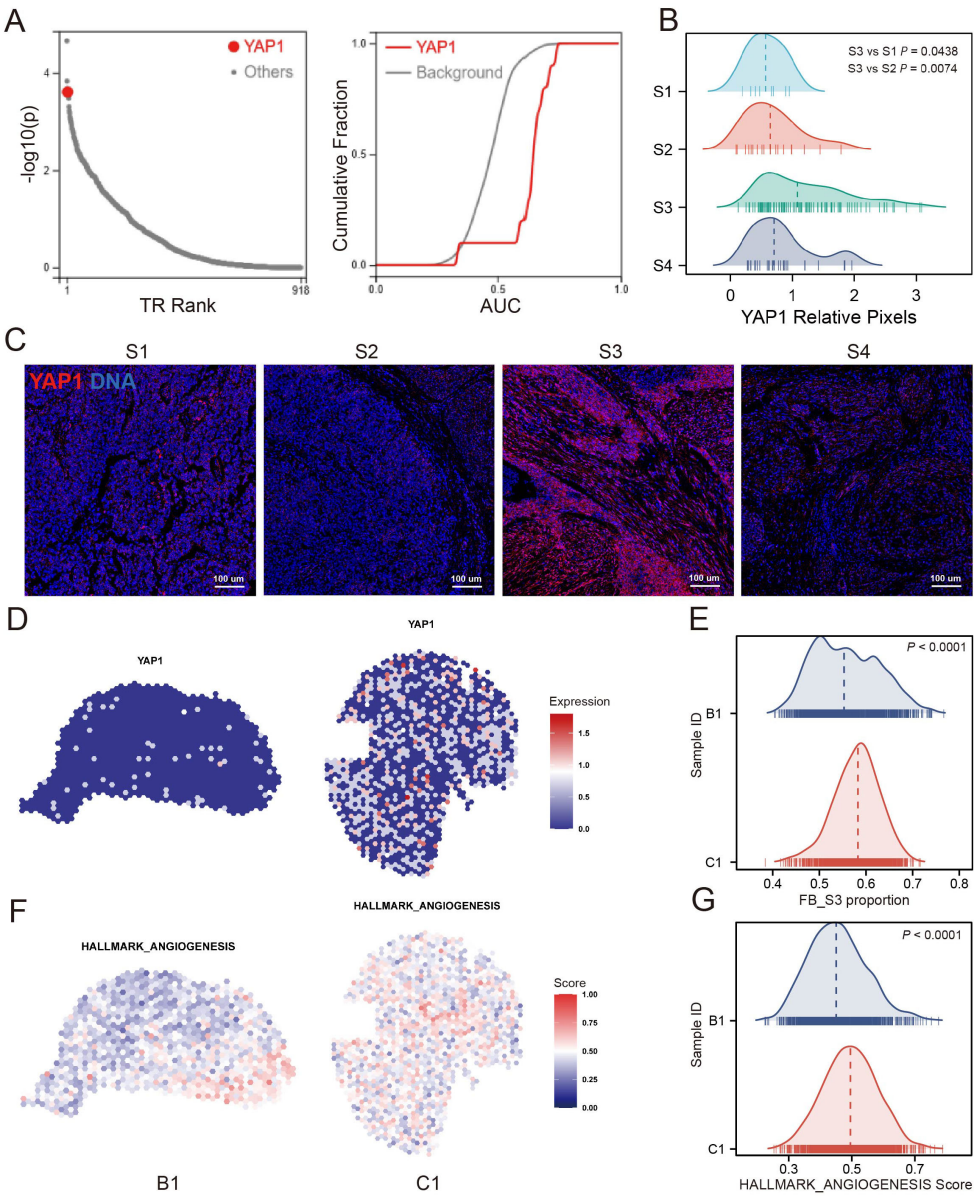
YAP1 was associated with the S3. **(A)** Transcription factor prediction analysis showed that YAP1 was one of the top potential transcription factors of S3 up-regulated gene. **(B)** YAP1 relative pixel intensity of FB cluster S1/S2/S3/S4 positive ROIs. **(C)** The representative images of FB cluster S1/S2/S3/S4 positive ROIs in which YAP1 shows as red, DNA shows as blue. **(D, E)** YAP1 expression of S3 enrichment sample (C1) and low-S3 enrichment sample (B1). **(F, G)** Angiogenesis signature score of S3 enrichment sample and low-S3 enrichment sample.

Further investigation into the regulatory mechanisms of S3, we employed the BART transcription factor prediction tool to analyze transcription factors associated with genes upregulated in S3 relative to other FB clusters. YAP1 emerged as one of the top predicted transcription factors (**Figure 6A**). IMC imaging confirmed high YAP1 expression in the microenvironment where S3 is located, contrasting with lower expressions in microenvironments dominated by FB S1, S2, and S4 (**Figures 6B, C**), suggesting a potential regulatory link between S3 and YAP1. To further determine the spatial characteristics and mechanisms associated with S3, we analyzed ST data from bladder cancer. ssGSEA results showed that S3-enriched samples showed higher angiogenesis signature scores, consistent with increased YAP1 expression compared with low-S3-enriched samples (**Figures 6D–G**). Altogether, these findings show that S3 may promote angiogenesis and could be regulated by YAP1.

### Construction of an S3-related prognostic signature based on machine learning

**Figure 7:**
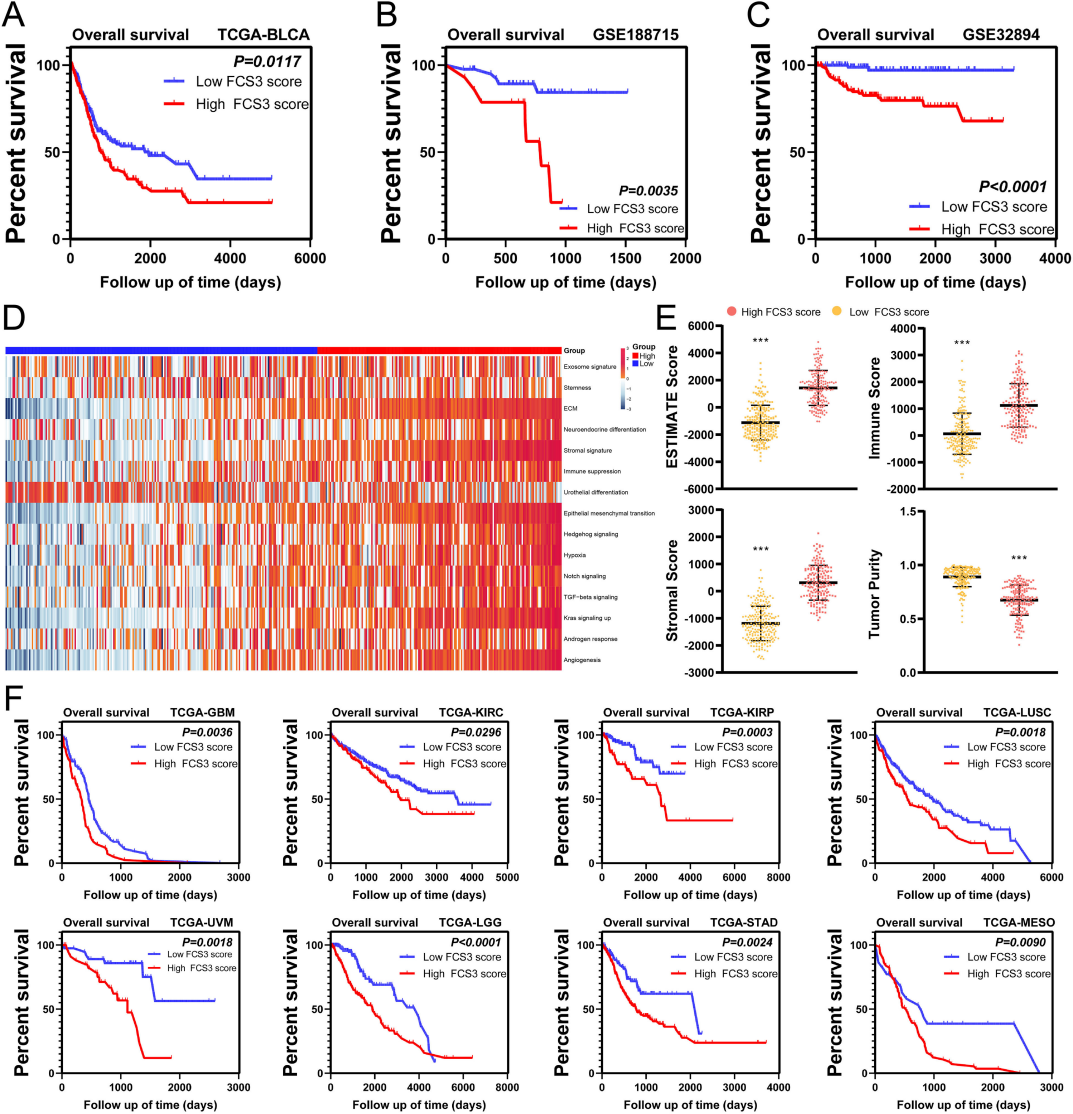
The fibroblast cluster S3 score (FCS3S) was associated with poor prognosis. **(A–D)** High FCS3S patients show poor over survival in TCGA-BLCA cohort **(A)**, GSE188715 cohort **(B)**, GSE32894 cohort **(C)**. **(D)** Heatmap of hallmark pathways. **(E)** Stromal score, immune score, tumor purity and ESTEMATE score in high/low FCS3S group in TCGA-BLCA cohort. (***P<0.001). **(F)** High FCS3S patients show poor over survival in glioblastoma multiforme (GBM), kidney renal clear cell carcinoma (KIRC), kidney renal papillary cell carcinoma (KIRP), lung squamous cell carcinoma (LUSC), uveal melanoma (UVM), lower-grade glioma (LGG), stomach adenocarcinoma (STAD), and mesothelioma (MESO).

To investigate the relationship between the presence of S3 and poor prognosis in MIBC, we utilized the XGBoost algorithm to analysis the gene expression profiles of S3 in scRNA-seq and construct an S3-related signature (**Supplementary Table S2**). Using this signature, we calculated the Fibroblast Cluster S3 Score (FCS3S) in the TCGA-BLCA cohort with the gene set variation analysis (GSVA) algorithm. Our results revealed that patients with high FCS3S had significantly poorer overall survival (**Figure 7A**). This finding was supported by two independent BLCA cohorts (GSE188715 and GSE32894) (**Figures 7B, C**). Subsequent analysis showed that patients with high FCS3S exhibited increased activation of pathways associated with malignant tumor progression, including angiogenesis, tumor stemness, cellular exosomes, immune suppression, extracellular matrix (ECM) remodeling, neuroendocrine differentiation, and epithelial–mesenchymal transition (EMT) in the TCGA-BLCA cohort, indicating a higher degree of malignancy in these patients (**Figure 7D**). ESTIMATE analysis showed that patients with high FCS3S had increased immune and stromal scores, as well as overall ESTIMATE scores, compared to those with low FCS3S (**Figure 7E**). Notably, the immune suppression score was significantly elevated in high FCS3S patients, suggesting greater immune suppression in this group (**Figure 7D**). Furthermore, we evaluated the prognostic value of FCS3S across various cancers and found that it can effectively identify patients with poor outcomes and the following eight cancer types: glioblastoma multiforme (GBM), kidney renal clear cell carcinoma (KIRC), kidney renal papillary cell carcinoma (KIRP), lung squamous cell carcinoma (LUSC), uveal melanoma (UVM), lower-grade glioma (LGG), stomach adenocarcinoma (STAD), and mesothelioma (MESO) (**Figure 7F**). To summarize, our findings verified that S3 is complicated and related to the progression of MIBC, and FCS3S serves as a robust tool for predicting patient outcomes across different cancer types.

## Discussion

The TME has garnered immense attention in cancer research. Recent advancements in scRNA-seq and cytometry by time-of-flight (CyTOF) have revealed unique phenotypic diversity and heterogeneity within the TME of MIBC (17, 21). However, a comprehensive understanding of the spatial landscape of the TME in MIBC has yet to be completely achieved. To address this gap, we performed a spatially resolved analysis that integrated IMC, scRNA-seq, and ST profiling. In the present study, IMC with 33 TME-associated markers was used, producing 6,105 highly multiplexed images from 40 patients. IMC allowed an in-depth analysis of spatial heterogeneity across the N, L, and T regions. We identified four distinct FB clusters in these regions, each with unique phenotypes and spatial distribution patterns. Furthermore, FB S3 (S3) was predominantly enriched in the L regions and closely associated with poor prognosis and angiogenesis. Subsequent investigations using scRNA-seq and ST analyses verified that S3 is complexly involved in angiogenic processes and interacted with endothelial cells via a unique L–R pair, NOTCH1–JAG2. Based on these aforementioned findings, we developed the FCS3S, a prognostic prediction model based on the characteristic genes of S3, which can accurately predict prognostic information in patients.

FB, the primary stromal cells in the TME, are crucial in collagen production (22). Many scRNA-seq studies have shown the presence of diverse fibroblast subpopulations within the TME, each characterized by novel molecular signatures (23, 24). In the present study, we used IMC to identify four distinct FB clusters in the TME of MIBC, each exhibiting different spatial distributions. Four unique types of FB were found in the T regions, whereas only specific types were present in the L and N regions. This heterogeneity indicates that each FB cluster may perform specific biological roles and respond differently to environmental stimuli. Furthermore, S3 emerged as the predominant FB localized in the L region—an area important for studying tumor invasion and metastasis, where tumor cells invade normal tissue and interact with stromal cells (25). The preferential localization of S3 within this active region indicates that it represents a highly active FB cluster, potentially arising from tumor cells training the resident fibroblasts. Survival analysis showed that patients with S3-positive tumors had significantly shorter overall survival compared with those with S3-negative tumors, highlighting the important role of S3 in the progression of MIBC. IMC analyses showed that relative to S3-negative tumors, S3-positive tumors exhibited higher vascular density, more extensive and morphologically complex blood vessels, and upregulated expression of VEGFR on the vessel walls, indicative of abnormal angiogenesis. Studies suggest that FB can secrete various factors, including VEGFA, PDGFC, FGF2, and osteopontin, which are known to stimulate angiogenesis in tumor tissues (26). Furthermore, FB facilitates angiogenesis by remodeling the ECM, affecting mechanical properties such as hardness, elasticity, and interstitial fluid pressure of the tumor stroma (27). They can also recruit endothelial progenitor cells to promote tumor neovascularization by secreting SDF1 (28). The tissue remodeling images obtained via IMC showed proximity between S3 and endothelial cells, further establishing an association between S3 and angiogenesis. Altogether, these findings suggest that S3 plays an important role in promoting abnormal angiogenesis in tumors.

The emergence of scRNA-seq has revealed the complexity of the TME of MIBC, underscoring the unique phenotypes within its cellular components. To determine the association between S3 and angiogenesis, we analyzed published scRNA-seq data from bladder cancer. Through clustering and cell annotation, we identified S3 at the transcriptomic level, consistent with previous characterization by IMC. Differential analysis showed that genes upregulated in S3 were significantly enriched in angiogenesis pathways compared with those with other FB clusters. L-R analysis showed that S3 exhibited the strongest interactions with endothelial cells, indicating a strong influence on angiogenic processes. To further elucidate the molecular mechanisms underlying the promotion of abnormal angiogenesis by S3, we performed an L–R analysis to determine specific pairs exclusive to S3 and endothelial cells. Nevertheless, this method, based solely on gene expression, lacks spatial context regarding cellular localization. To confirm our findings, we explored publicly available ST data for bladder cancer. Analysis of spatial gene expression patterns within the tissue identified a notable colocalization of NOTCH1–JAG2, a distinct L–R pair between S3 and endothelial cells. Pietras et al. reported that JAG2 can activate the NOTCH signaling pathway under hypoxic conditions to promote endothelial cell tube formation (29), elucidating a mechanism by which S3 may increase abnormal angiogenesis via the NOTCH1–JAG2 interaction.

Further analysis to elucidate the regulatory mechanisms of S3 involved a transcription factor prediction for genes significantly upregulated in S3. YAP1 was identified as the third-ranked transcription factor. YAP1 is a major mediator in signaling pathways associated with tumorigenesis and exhibits widespread activation in malignant human tumors (30). Recent a study has reported the close relationship between YAP1 and FB activation, emphasizing the role of YAP1 in influencing FB behavior (31). Bora–Singhal et al. reported that YAP1 can increase tumor angiogenesis by upregulating VEGFA expression (32). Our IMC-based tissue remodeling analysis further showed significantly upregulated YAP1 expression within the microenvironment of S3, both in stromal and tumor cells, compared with other FB clusters. Altogether, these findings strongly suggest that S3 may be intricately regulated by YAP1 signaling pathways, contributing to its proangiogenic properties.

To investigate the prognostic role of S3, we used machine learning algorithms to identify the characteristic genes associated with S3. Using the GSVA algorithm, we assigned a prognostic score based on these genes to each patient. Our findings showed that the FCS3S effectively identified patients with unfavorable prognoses across various cancer types, including MIBC, MESO, GBM, LGG, STAD, UVM, KIRC, KIRP, and LUSC. Patients in the TCGA-BLCA cohort with high FCS3S levels exhibited higher stromal and immune scores than those with low FCS3S levels. They also showed upregulated expression of genes related to malignant tumor progression, such as angiogenesis, tumor stemness, and immune suppression. These findings suggest that FCS3S is significantly associated with tumor progression, and patients with high FCS3S levels may respond to antiangiogenic therapies and immunotherapy. Presently, anti-VEGF drugs, including sunitinib, axitinib, and sorafenib are approved for treating advanced and metastatic tumors. However, the development of resistance to anti-VEGF therapy poses considerable challenges (33). Combining antiangiogenic agents and immune checkpoint inhibitors may have synergistic effects in promoting tumor vessel normalization and stimulating immune activation (34). Previous studies indicate that tumor vessel normalization can improve the aggregation of immune cells and boost immune function, whereas immune cell activation reciprocally promotes vessel normalization. Their combined application establishes a positive feedback loop, thereby exhibiting potent antitumor effects (35). Furthermore, in patients with high FCS3S, a promising approach can involve combination therapy using anti-VEGF drugs alongside immunotherapy, leading to more favorable clinical outcomes.

To conclude, we described the spatiotemporal heterogeneity of the microenvironment in MIBC, underscoring the unique spatial structure of the N, L, and T regions, and the enrichment of the specific S3 at the L regions. This FB cluster was closely associated with adverse prognoses in MIBC, playing an important role in promoting aberrant tumor angiogenesis via the modulation of the NOTCH1–JAG2 L–R pair. Based on these insights, we established an S3-associated prognostic signature, which can be effective in the prognostic evaluation of patients with any nine different cancer types. Altogether, our findings provide novel insights into the TME of MIBC, offering novel opportunities and targets for the personalized treatment of MIBC.

## Data Availability

The original contributions presented in the study are included in the article/**Supplementary Material**. Further inquiries can be directed to the corresponding authors.

## Supplementary material

Supplementary Figure 1 The case data of the 40 MIBC patients.

Supplementary Figure 2 The cell number of per ROI.

Supplementary Figure 3 ScRNA-seq analysis revealed the diversity phenotype of FB in MIBC. **(A)** tSNE plot of 35 cell cluster in MIBC. **(B)** tSNE plot of several cell types. **(C)** tSNE plot of 14 FB clusters. **(D)** The FB clusters exhibited different gene expression patterns.
