# Supplementary figures and images for: Spatially-resolved analyses of muscle invasive bladder cancer microenvironment unveil a distinct fibroblast cluster associated with prognosis
Source: Front Immunol. 2024 Dec 20;15:1522582. doi: 10.3389/fimmu.2024.1522582 (PMC11695344; doi:10.3389/fimmu.2024.1522582)

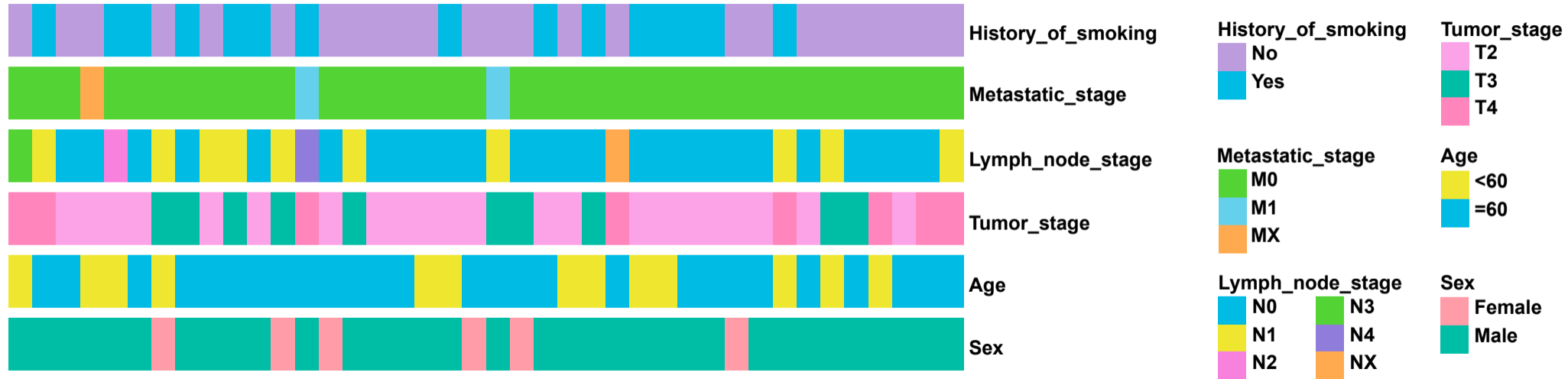

Supplement: Supplementary Figure 1 — The case data of the 40 MIBC patients. [file DataSheet1.pdf]

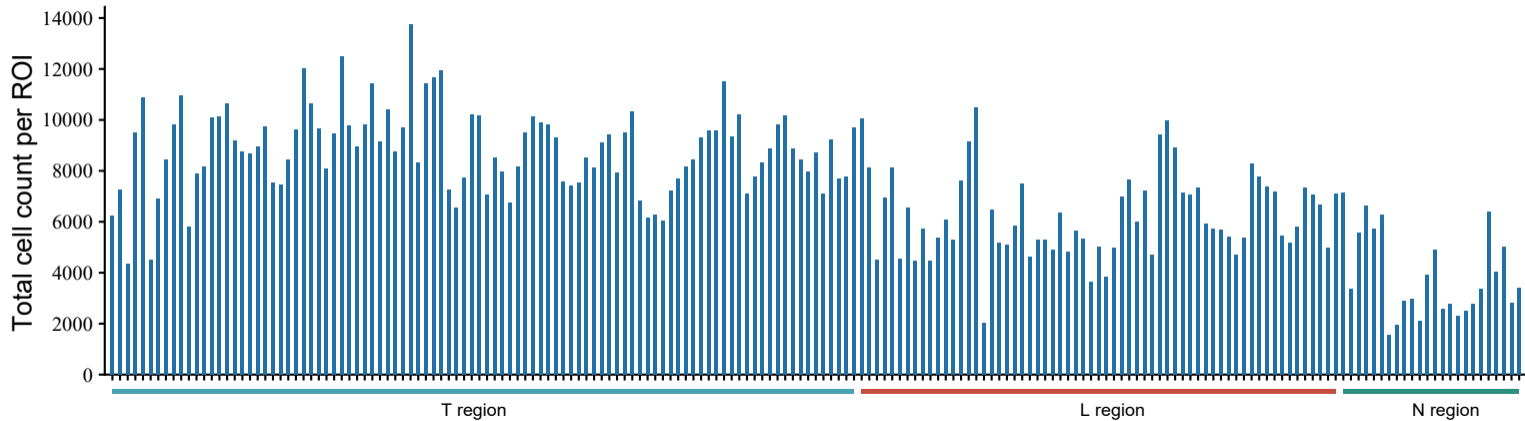

Supplement: Supplementary Figure 2 — The cell number of per ROI. [file DataSheet2.pdf]

**A****All cells**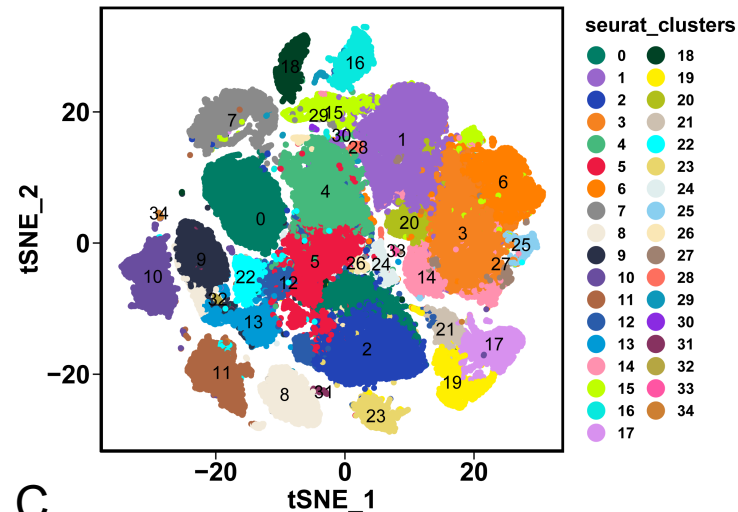**B****All cells**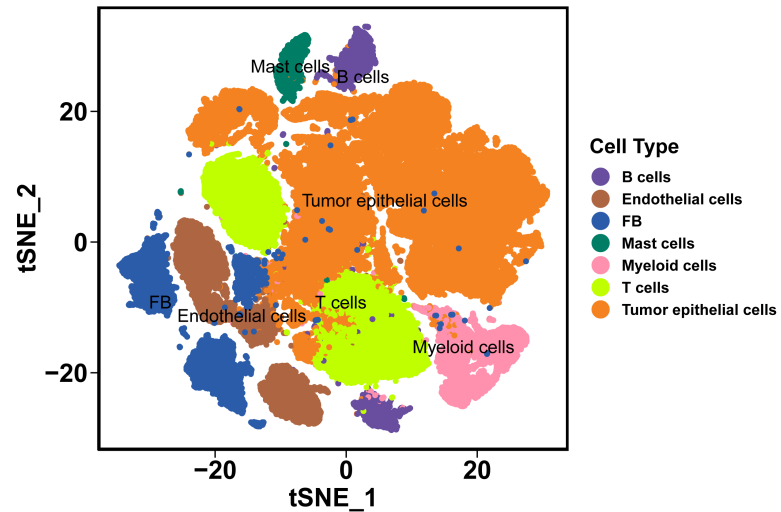**C****FB**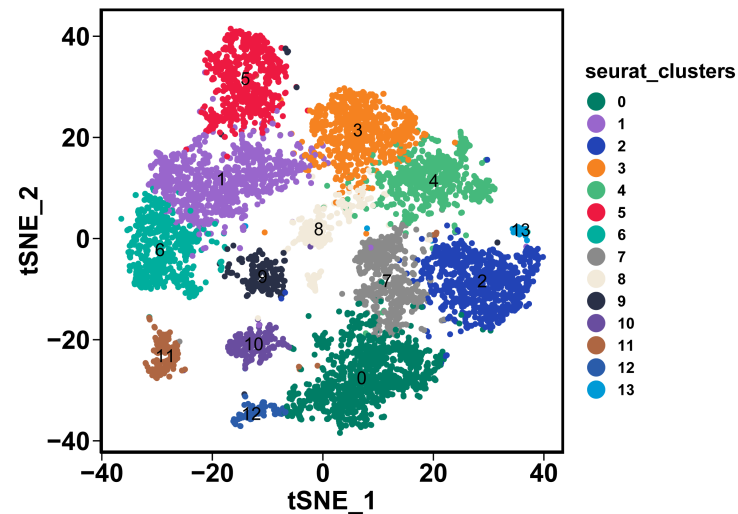**D****ScRNA-seq**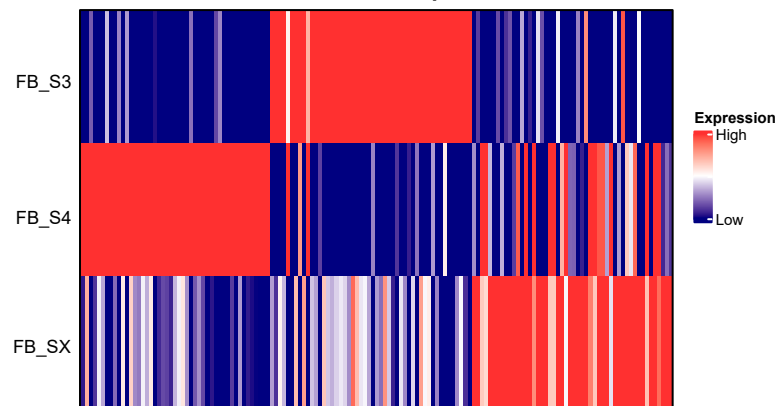

Supplement: Supplementary Figure 3 — ScRNA-seq analysis revealed the diversity phenotype of FB in MIBC. (A) tSNE plot of 35 cell cluster in MIBC. (B) tSNE plot of several cell types. (C) tSNE plot of 14 FB clusters. (D) The FB clusters exhibited different gene expression patterns. [file DataSheet3.pdf]
