# Supplementary material for: Spatially-resolved analyses of muscle invasive bladder cancer microenvironment unveil a distinct fibroblast cluster associated with prognosis
Source: Front Immunol. 2024 Dec 20;15:1522582. doi: 10.3389/fimmu.2024.1522582 (PMC11695344; doi:10.3389/fimmu.2024.1522582)
Supplement: Supplementary file 4 [file DataSheet4.pdf]

**Table S1. antibodies information of IMC**

| Antibodies | Label /Metal | Source/ Company | Product_ID /Identifier |
|---|---|---|---|
| FAP | 147Sm | Abcam | ab53066 |
| CD31 | 156Gd | Abcam | ab9498 |
| VEGFR | 175Lu | Abcam | ab39638 |
| E-cadherin | 158Gd | Fluidigm | 3158029D |
| Vimentin | 143Nd | Fluidigm | 3143029D |
| YAP1 | 149Sm | Abcam | ab56701 |
| CD66a | 171Yb | Fluidigm | 3171020D |
| Ki-67 | 168Er | Fluidigm | 3168022D |
| CK-5 | 164Dy | Abcam | ab53121 |
| SNAIL+SLUG | 151Eu | Abcam | ab180714 |
| ER-β | 174Yb | Abcam | ab288 |
| CD133 | 172Yb | Abcam | ab16518 |
| Foxp3 | 155Gd | NOVUS | NB100-39002 |
| PD_L1 | 145Nd | Abcam | ab226766 |
| AR | 166Er | Abcam | ab9474 |
| CTLA4 | 173Yb | Abcam | ab237712 |
| PD-1 | 154Gd | Abcam | ab201811 |
| ALDH | 146Nd | BD | 611195 |
| CD47 | 144Nd | R&D | AF4670 |
| TGF-β1 | 167Er | Abcam | ab190503 |
| CD68 | 141Pr | Abcam | ab213096 |
| CD4 | 159Tb | Biolegend | 300502 |
| CD8a | 162Dy | Fluidigm | 3162034D |
| CD20 | 161Dy | Fluidigm | 3161029D |
| CD3 | 170Er | Fluidigm | 3170019D |
| CD45 | 152Sm | Fluidigm | 3152016D |
| Alpha-SMA | 142Nd | Abcam | ab5694 |
| Beta-catenin | 165Ho | Fluidigm | 3165032D |
| CD90 | 163Dy | Abcam | ab181469 |
| Collagen I | 169Tm | Fluidigm | 3169023D |
| Pan-CK | 160Gd | Abcam | ab7753 |
| CD326/EpCAM | 150Nd | Abcam | ab71916 |
| CD44 | 153Eu | Abcam | ab157107 |
