# Supplementary material for: Spatially-resolved analyses of muscle invasive bladder cancer microenvironment unveil a distinct fibroblast cluster associated with prognosis
Source: Front Immunol. 2024 Dec 20;15:1522582. doi: 10.3389/fimmu.2024.1522582 (PMC11695344; doi:10.3389/fimmu.2024.1522582)
Supplement: Supplementary file 5 [file DataSheet5.pdf]

Table S2 The fibroblast cluster S3-related signature

ARHGDIB	MYH9	NPC2	COL1A2	CXCL14
THY1	LOXL2	CEBPB	CD59	S100A6
MARCKS	CCL5	NREP	C1S	RGS5
CCL21	ADAM12	RGS16	MT2A	MARCKSL1
CALD1	CD3D	RPS19	IGFBP4	CFL1
CFD	DCN	FOS	SERF2	SH3BGRL3
ANGPT2	TMSB10	CCL19	HLA-DRB1	ARPC2
SPARC	STEAP4	CD52	LGALS1	HLA-DRA
ADGRF5	MYH11	MFAP4	FTL	DNAJA1
FBLN1	GMFG	MYL9	MGP	APOD
